# Evaluation of 7q31 region improves the accuracy of *EGFR *FISH assay in non small cell lung cancer

**DOI:** 10.1186/1746-1596-4-36

**Published:** 2009-11-04

**Authors:** Laura Casorzo, Mara Corigliano, Paolo Ferrero, Tiziana Venesio, Mauro Risio

**Affiliations:** 1 Unit of Pathology. Institute for Cancer Research and Treatment, Strada Provinciale 142, 10060 Candiolo-Torino, Italy

## Abstract

**Background:**

Increase of *EGFR *gene copy number consequent to gene amplification and/or polysomy of chromosome 7 has been significantly associated with better clinical outcome in Non Small Cell Lung Cancer (NSCLC) patients treated with Tyrosin-Kinase Inhibitors (TKIs).

The primary method to detect *EGFR *copy number is FISH (Fluorescence in Situ Hybridization), that in lung cancer requires a precise standardization due to the presence of intratumor heterogeneity and high frequency of chromosome 7 polysomy.

Recommendations and interpretative guidelines to discriminate NSCLC patients into FISH positive (gene amplification and high chromosome 7 polysomy) and FISH negative have been proposed by the University of Colorado Cancer Center (UCCC). However, in a subset of cases the distinction between *EGFR *amplification and chromosome 7 polysomy can be controversial because of a complex pattern of multiple *EGFR *and centromere signals.

**Methods:**

In order to distinguish more accurately these two genetic events, 20 NSCLC FISH positive patients, showing a controversial pattern of *EGFR *and centromere specific signals, were further evaluated for the status of 7q31 distal region.

**Results:**

A discrepancy between FISH results obtained with UCCC scoring system and 7q31 control was evidenced in 2 patients (10%).

**Conclusion:**

Our data strengthen the usefulness of 7q31 region evaluation to discriminate EGFR amplification from chromosome 7 polysomy in controversial *EGFR *FISH positive cases. Since it has been reported a possible different contribution of amplification and polysomy to TKIs susceptibility in NSCLC, the clear distinction between these two genetic events may be important to identify a subset of patients more responsive to the therapy.

## Background

The development and clinical application of Tyrosine Kinase Inhibitors (TKIs) targeting the Epidermal Growth Factor Receptor (EGFR), such as erlotinib and gefitinib, provide important insights for the treatment of non small cell lung cancer (NSCLC). However, patients derive different degrees of benefit from treatment with EGFR TKIs [1-3]. Several molecular studies showed that sensitivity to EGFR TKIs correlated very strongly with a class of somatic activating mutations of EGFR kinase domain that target mainly two exons (19 and 21), indicating that patients harbouring *EGFR *mutations have a higher partial and complete response rate to EGFR TKIs therapy than those without mutations [4-6]. Overall, the incidence of *EGFR *mutations in NSCLC among clinical responders to gefitinib or erlotinib is 77%, but this is not always associated with prolonged survival [7-11]. However, approximately 10-20% of patients who show a partial response to EGFR TKIs have not detectable *EGFR *mutations, suggesting other molecular mechanisms are involved in TKIs response [9].

Although a conclusive picture has yet to emerge, an association between increased *EGFR *gene copy number, detected by Fluorescence in Situ Hybridization (FISH), and sensitivity to TKIs therapy was observed. Cappuzzo et al. [12] reported that FISH positive patients showed a better response and an increased survival in respect to FISH negative patients (36% *vs *3% and 18 months *vs *7 months, respectively). Different studies [13-18] demonstrated a significant association between high *EGFR *copy number and response to gefitinib, time to progression and survival. In addition, it was shown that patients negative for at least two tests among mutation, FISH and immunohystochemistry had no benefit from TKI therapy [19].

According to the Molecular Assays in NSCLC Working Group, FISH represents the gold standard method to establish gene numerical status combining the gene specific probe and the control centromeric probe [20]. FISH allows to detect the real amount of gene copies in relation to the chromosome number and discriminate between gene amplification from high gene copy number due to polysomy. Normally amplification is expressed as gene copy number/chromosome control number ratio of > 2.

In NSCLC high *EGFR *gene copy number results mostly from chromosome 7 polysomy while less often from gene amplification. In the first case, the high number of chromosome 7 centromeres lowers the gene/chromosome ratio value to less than 2, masking possible amplification events. Moreover, *EGFR *gene is located at the band 7p12, very close to the centromere; as a consequence, in a subset of tumors amplification involves *EGFR *gene and centromeric sequences too, originating a complex pattern of multiple red and green signals difficult to classify as amplification or high polysomy.

Recently, a FISH scoring system and guidelines for *EGFR *FISH assay in NSCLC patients have been proposed by the University of Colorado Cancer Center (UCCC). This system stratifies results into six groups by number of copies of the *EGFR *gene, including disomy, low trisomy, high trisomy, low polysomy, high polysomy and gene amplification. High polysomy and gene amplification are considered FISH positive, the cut-off being assumed on a retrospective study performed in advanced NSCLC patients treated with gefitinib [21]. Although the scoring criteria of UCCC for stratification of NSCLC patients with *EGFR *FISH assay effectively copes with complexity of *EGFR *gene amplification mechanisms, it doesn't always allow to discriminate between real gene amplification and high level polysomy, particularly when almost the same number of centromere 7 and *EGFR *gene signals is present.

The aim of the study was to improve the discrimination between amplification and high level polysomy in those controversial cases that are characterized by multiple copies of both *EGFR *gene and centrome. To this purpose, we have introduced the FISH evaluation of a further chromosome 7 region (7q31), sufficiently far from *EGFR *locus (7p12), in 20 selected FISH positive patients showing a complex pattern of *EGFR *and centromere signals.

## Materials and methods

### Patients and samples

The study was conducted on 20 NSCLC patients selected over 90 (median age: 69; range 44-87 years) with cytologically or hystologically confirmed NSCLC in advanced stage (III-IV), according to WHO criteria: 18 adenocarcinoma, 1 mucoepidermoid carcinoma and 1 squamous cell carcinoma. The majority were females (14/20; 70%) and never smokers (16/20; 80%). Eleven patients (55%) had a history of metastasis. Mutations of EGFR gene were present in 18/20 patients (90%) who, consequently, received therapy with erlotinib. Ten patient had exon 19 deletion, 7 had mutation of exon 21 (6 cases with substitution L2573 T>G and 1 case with substitution L2558 T>C) and 1 had exon 20 deletion.

All patients had been found *EGFR *FISH positive for amplification or high level polisomy in a preliminary analysis. They were selected for this study because of a high number of both *EGFR *and centromere signals in the absence of peculiar traits of gene amplification, such as *EGFR*/CEP 7 ratio >2, or the presence of large or small clusters of signals. Such a picture is particularly difficult to interpret because it might reflect either an amplification involving EGFR gene together with centromeric sequences or, alternatively, a polysomy of chromosome 7.

The preliminary FISH analysis was carried out using LSI *EGFR*/CEP 7 (Vysis-Abbott). According to UCCC criteria, high level polysomy was defined as the presence ≥ 4 *EGFR *copies in > 40% of cells, and gene amplification as: a) *EGFR*/centromere 7 ratio ≥ 2 b) presence of small gene clusters (4-10 copies) in ≥ 10% of the tumor cells c) larger and brighter *EGFR *signals than expected in >10% of the tumor cells d) ≥ 15 copies of the *EGFR *signals in ≥ 10% of the tumor cells.

The evaluation of 7q31 region was performed by FISH on paraffin embedded specimens derived from 18 primary tumors and 2 secondary lesions (lymph node and bone). These specimens included 2 tissue sections from surgical lobectomy, 6 fine needle aspiration/core biopsies (FNAB), 3 endoscopic bronchial biopsies, 3 bronchial washings and 6 pleural effusions.

In 10 cases the median number of *EGFR *gene (*EGFR *gene/nuclei ratio) and the median number of chromosome 7 centromere (centromere 7/nuclei ratio) were very similar with a Δ < 1 unit. In the other 10 cases the median number of *EGFR *gene was higher than the median number of chromosome 7 centromere that, nevertheless, ranged from 6 to 23 copies.

### 7q31 FISH Analysis

**Figure 1:**
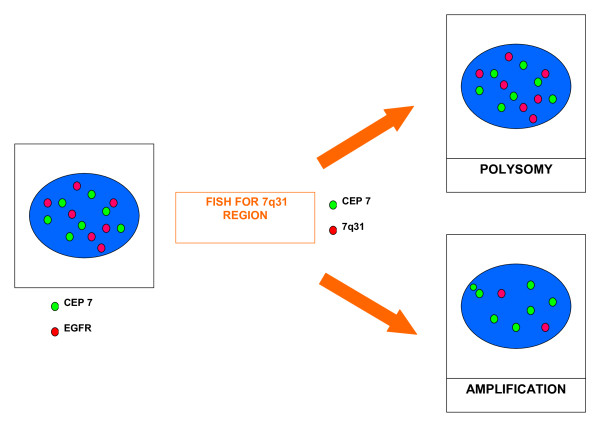
**EGFR/CEP7 co-amplification or Polisomy of chromosome 7?**. In case of polysomy, the copy number of 7q31 region increases similarly to centromere signals. EGFR high copy number in the presence of an eusomic status of 7q31 region is indicative of gene amplification.

FISH analysis was performed using standard methods. In brief, 4-6 μm paraffin embedded tissue sections were deparaffinized and subsequently enzymatically digested with a commercial kit (Paraffin pretreatment reagent kit, Vysis-Abbott Molecular, Downers Grove, IL, USA). Sample DNA was denaturated at 75°C for 5 minutes. Spectrum-Orange-labeled LSI D7S522 probe, that hybridises to band 7q31, was used together with Spectrum-Green-labelled centromere 7 reference probe (LSI D7S522/CEP 7, Vysis-Abbott). The probe mix (10 μl), previously denaturated for 5' at 75°C, was applied on each slide. The slides, covered with a glass coverslip, were incubated for 5' at 79° for codenaturation and placed in a humidified chamber at 37°C overnight to allow hybridisation to occur. After post-hybridization washes, carried out according to manifacturer's protocols, tissue sections were counterstained with DAPI I (Vysis-Abbott) and examined with a fluorescence microscope (Olympus, Tokyo, Japan). An average of 40 non-overlapping nuclei with intact morphology were analysed using H&E-stained sections as hysto-topographic reference. Copy number of 7q31 specific signals was estimated for each tumor sample and the median number of 7q31 band signals (7q31 number/nuclei) was calculated for each patient. Particular attention was focused on those nuclei exhibiting high number of chromosome 7 centromeres. If the copy number of 7q31 region was not increased similarly to chromosome 7 centromere, polysomy could be excluded; conversely, increased 7q31 signals paralleling the increased centromere signals was considered as a signature of polysomy (Fig. 1).

## Results

According to UCCC scoring system, 13 out of 20 patients (65%) showed high level polysomy. Seven patients (35%) were classified as amplified cases, because of the presence of *EGFR*/CEP7 ratio >2 (cases n° 4 and 20), tight gene clusters and larger and brighter *EGFR *signals in > 10% of cells (n° 5 and 19) and of >15 *EGFR *signals in ≥ 10% of cells (n° 2, 7 and 13).

Using 7q31 band FISH evaluation, almost the same mean number of 7q31 band and chromosome 7 centromeres was observed in 10 cases (n° 3, 5, 6, 9, 14, 15, 16, 17, 18, 19), confirming a polysomic status. In cases n° 5 and 19, however, a cell population with amplification was co-present. In 4 cases (cases n° 4, 7, 10, 13) 7q31 mean number was lower than centromere mean number as expected in case of gene amplification. Patients n° 2 and 11 who had very similar value for *EGFR *gene and 7q31 band, even higher than centromere number, were considered polysomic. Patients n° 1, 8, 12, because of their relatively low *EGFR *copy number, were supposed to be polysomic even if 7q31 mean number was lower than centromere mean number. In these cases the presence of complex chromosomal rearrangements, resulting in chromosome 7 long arm losses and short arm gains, was more conceivable.

The results obtained applying UCCC criteria alone and UCCC criteria together with 7q31 FISH control were in agreement in 18/20 cases (90%). Case n° 2, classified as amplified having 10% of cells with >15 *EGFR *copies, had to be defined polysomic because of the presence of about the same number of 7q31 band and *EGFR *gene. In case n° 10, according to UCCC protocol, the presence of nuclei with *EGFR *gene high number (>15) confined just in 2,7% of cells suggested chromosome 7 polysomy rather than amplification. The eusomic status of 7q31 region (7q31/nuclei ratio = 2.2), much lower than centromere mean number, might be the consequence of chromosome 7 long arm deletion, resulting in 7q31 loss.

**Figure 2:**
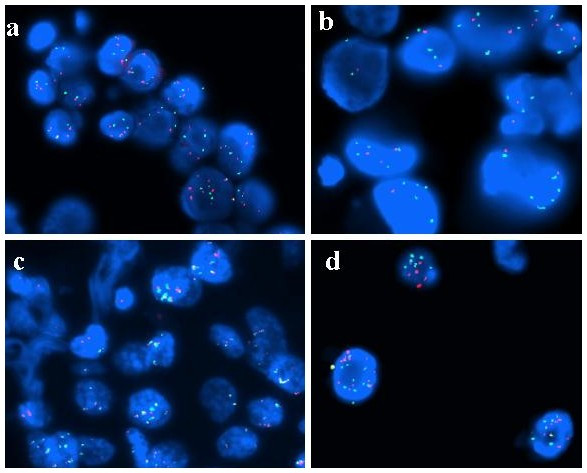
**FISH analysis of 7q31 region**. Case n° 10: a) FISH in bronchial biopsy section showing gain of both *EGFR *gene and chromosome 7 centromere; b) the patient is classified as amplified on the basis of eusomic status of 7q31 region. Case n° 2: c) FISH in pleural effusion sample with EGFR and centromere balanced aneusomy; d) increased copy number of 7q31 region is a signature of polysomy.

However, the presence of EGFR increased copy number with a percentage of cells harbouring >15 EGFR signals (even <10% of neoplastic population), together with a high number of chromosome 7 centromeres and a very low number of 7q31 region, suggested a gene amplification rather than chromosome 7 polysomy associated with deletion 7q31 (Fig. 2).

Results obtained with UCCC criteria alone and UCCC criteria with 7q31 FISH control are compared in Table 1.

**Table 1:** FISH results according to UCCC criteria and 7q31 control

Case N°	EGFR/CEP7 Ratio	EGFR/NUCLEI Ratio (Range)	CEP7/NUCLEI Ratio(Range)	% of cells with >4 EGFR copies	NOTES	RESULTS (UCCC criteria)	7q31/NUCLEI Ratio (Range)	CEP7/NUCLEI Ratio (Range)	RESULTS (7q31 control)
1	1.13	5.35 (3-8)	4.7 (2-8)	85%		P	2.02 (1-4)	4 (2-8)	P

2	1.50	8.4 (4-17)	5.55 (2-10)	100%	10% of cells with >15 EGFR copies	**A**	8.58 (4-14)	7.2 (2-13)	**P**

3	1.09	4.48 (3-9)	4.1 (2-9)	89%		P	3.97 (2-5)	4.8 (2-7)	P

4	2.80	12.42 (5-23)	4 (2-7)	100%	EGFR/CEP7 Ratio >2	A	2(2)	5.3 (4-8)	A

5	1.50	7.4 (3-16)	4.9 (2-8)	100%	22,5% of cells with tight gene clusters	A	4.48 (2-9)	4 (2-7)	A

6	1.45	6.2 (3-12)	4.2 (2-8)	84%		P	4.44 (3-7)	4.72 (2-7)	P

7	1.0	15.2 (10-40)	14.5 (8-35)	100%	80% of cells with >15 EGFR copies	A	1.70	18 (12-45)	A

8	1.16	5.58 (3-10)	4.78 (3-9)	97%		P	2.37 (1-4)	5.2 (4-10)	P

9	1.05	4.92 (2-11)	4.64 (2-9)	82%		P	5.07 (2-9)	6.7 (2-11)	P

10	1.69	8.52 (4-22)	5.04 (2-9)	97%	2.4% of cells with >15 EGFR copies	**P**	2.2 (1-4)	5 (2-12)	**A**

11	1.24	4.89 (2-10)	3.93 (2-8)	75%		P	6.07 (3-12)	6 (2-10)	P

12	1.32	7.96 (3-15)	6.01 (3-9)	97%	2,7% of cells with >15 EGFR copies	P	3.2 (1-6)	6 (3-10)	P

13	1.25	10.5 (4-26)	8.36 (4-23)	100%	16% of cells with >15 EGFR copies	A	2.6 (2-5)	7.7 (4-18)	A

14	1.06	5.95 (2-13)	5.6 (2-12)	100%		P	4.1 (2-5)	6.3 (2-11)	P

15	1.18	4.39 (2-13)	3.72 (1-13)	72%		P	3.36(2-5)	3.9 (2-10)	P

16	1.22	4.64 (2-9)	3.78 (2-6)	71%		P	2.35 (1-3)	4.2 (2-7)	P

17	1.70	3.9 (1-8)	2.2 (1-5)	55%		P	2.24 (1-4)	3 (2-6)	P

18	1.10	4.9 (2-8)	4.4 (2-7)	90%		P	6.02 (4-9)	7.4 (2-10)	P

19	1.40	6.4 (3-23)	4.7 (1-10)	93%	Tight gene clusters in 11,5% of cells	A	4.15 (2-7)	5.6 (2-10)	A

20	2.90	8.8 (2-13)	3.1 (2-6)	88%	EGFR/CEP7 Ratio >2	A	1.6 (1-3)	2.1 (2-5)	A

A = Amplification; P = Polysomy

## Discussion

The development of small molecule inhibitors of EGFR resulted in new therapeutics options for patients with advanced NSCLC. Clinical trials provide sufficient evidence that identification of molecular markers for predicting the effects of TKIs may become an integral part of the future standard of care for patients with NSCLC [22-27]. As a consequence, standardized assay procedures must be developed.

Although the significance is still controversial [28-30], *EGFR *copy number may be predictive of response to EGFR TKIs therapy and, presently, FISH is the gold standard method to detect *EGFR *gene numerical status. Normally, the gene specific probe is used coupled with a centromeric probe as control to assist in distinguishing gene amplification from chromosomal polysomy. Increased gene copy number, in association with an increase in the number of the chromosome the gene maps on, is interpreted as polysomy. However, FISH analysis in NSCLC is complicated by multiple factors. Lung cancers are frequently heterogeneous with significant variability of *EGFR *copy number within the same tumor. Moreover, in NSCLC high *EGFR *gene copy number results more often from chromosome 7 polysomy rather than gene amplification. This depends on the high level of chromosomal instability, as demonstrated by the extremely complex karyotypes. The modal chromosome numbers usually cluster in the near-triploid to near-tetraploid range and the most frequent numerical alteration is polysomy 7 or 7p, with the number of 7/7p exceeding the number of copies expected based on the ploidy of the tumor. Even among the structural rearrangements, the most frequently affected band is 7p11-12, where *EGFR *gene maps [31-33]. Furthermore, *EGFR *gene is located at the band 7p12 very close to centromere and *EGFR *amplicon may include centromeric sequences too, originating a coamplification-type gene amplification, with a complex pattern of signals. Finally, polysomy may coexist with gene amplification, giving rise to a high number of both centromere and *EGFR *signals.

A useful FISH scoring system for *EGFR *FISH evaluation in NSCLC patients has been introduced by the University of Colorado Cancer Center (UCCC) [21]. This system stratifies results into six groups (disomy, low trisomy, high trisomy, low polysomy, high polysomy and gene amplification) according to the number of copies of the *EGFR *gene, chromosome 7 centromere and their frequency in tumor cells of the sample. High polysomy and gene amplification are considered FISH positive.

The guidelines suggested by the Molecular Assays in NSCLC Working Group recommend a two color FISH with centromeric probe as control and encourage the scoring system described by the Colorado group to be used for FISH assay interpretation. Nevertheless authors invite other investigators to propose algorithms for *EGFR *FISH evaluation [20].

In our study, we introduced a further FISH control, analysing chromosome 7 distal region (7q31) in a series of 20 FISH positive patients with advanced NSCLC and a complex pattern of signals. Fair concordance was seen in 18/20 (90%) cases. However in 2 cases (10%) we found discrepancy between FISH results obtained with UCCC scoring system and 7q31 FISH control. According to UCCC criteria, patient n° 2, because of the presence of 10% of cells with >15 *EGFR *gene copies, was considered amplified. On the contrary, FISH evaluation of 7q31 region revealed almost the same number of both 7q31 and centromere signals (8,4 vs. 8,58), showing that the *EGFR *gene increased copy number is consequent to chromosome 7 polysomy. Patient n° 10, having <10% of cells with >15 *EGFR *gene copies, was classified as polysomic. However, the eusomic status of 7q31 band suggested the presence of *EGFR *gene amplification. Our results show that in controversial cases, characterized by multiple *EGFR *and centromere specific signals, 7q31 FISH detection increases accuracy in discriminate *EGFR *amplification from chromosome 7 polysomy.

From the molecular point of view, amplification and polysomy are different genetic events. Amplification is an alteration by which a cell acquires multiple copies of a DNA sequence, the size ranging from a few hundred kilobases to megabases and is one of the mechanisms by which protooncogenes may be activated in tumor cells. In that case chromosomes may display homogeneously staining regions or small acentric circular autonomously replicating double minutes. Polisomy, reflecting genetic instability, may arise from mitotic non-disjunction followed by secondary endoreduplication events, and deregulate global gene transcription in cancer cells.

In contrast to breast cancer, where *HER2 *amplification, but not chromosome 17 polysomy seems to confer sensitivity to monoclonal antibody trastuzumab [34,35], it has been reported that patients with NSCLC can derive a clinical benefit from TKIs therapy when *EGFR *increased copy number (ICN) is found, without distinction between amplification and/or high level of chromosome 7p polisomy. However, recent *in vitro *and clinical studies, focused on the relationship between mutations and ICN of *EGFR *gene, have shown that mutations are preferentially associated with amplification of the gene. The observation that four of six human NSCLC cell lines harbouring *EGFR *mutations were positive for *EGFR *amplification, whereas none of the ten cell lines negative for *EGFR *mutations manifested *EGFR *amplification, supported a close association between these two genetic alterations [36]. As far as clinical studies, Nomura et al (2007) observed a close relationship between three polymorphisms of *EGFR *gene, activating mutations, and selective amplification of the mutant allele, suggesting that just mutation promotes and drives mechanisms of amplification [37]. Similar evidence was reported in glioblastomas, where the variant form of *EGFR *gene frequently demonstrates allele-specific amplification [38,39]. Recent studies have reported that *EGFR *mutations occur early during multistage pathogenesis of NSCLC and precede amplification, but not necessarily high polysomy [37,40,41]. In addition, Morinaga et al (2008) observed a specific association between mutations and amplification, underlying that *EGFR *amplification, but not high polysomy, correlates with mutations [42].

As a consequence, a higher sensitivity and specificity of the *EGFR *FISH assay can turn out in an improved identification of those subsets of patients who can benefit more from TKI therapy.

We are currently investigating a wider cohort to characterize furtherly the relationship among ICN, mutations and TKI response. Preliminary data, regarding 16 *EGFR *mutated patients, indicate that the median overall survival is 16 months in presence of an *EGFR *amplification *vs *9.5 months in cases associated with 7p polysomy (unpublished data).

In conclusion, as it is conceivable that amplification and polysomy differently affects *EGFR *expression, activation, and sensitivity to TKIs, we underlie the importance of discriminating these two genetic events with accuracy.

## Conclusion

Results from this study strengthen the applicability and usefulness of the scoring criteria developed at UCCC for the assessment of *EGFR *gene patterns, however the further control of a different and distal region of chromosome 7, specifically 7q31, adds more accuracy in interpreting controversial FISH results, when multiple centromeric and gene specific signals are present. Distinction between *EGFR *gene amplification and high chromosome 7 polysomy is required, till the role of increased *EGFR *copy number in NSCLC patients in contributing to lung cancer oncogenesis and susceptibility to TKIs will be definitely established.

## Abbreviations

(FISH): Fluorescence in Situ Hybridization; (NSCLC): Non Small Cell Lung Carcinoma; (EGFR): Epidermal Growth Factor Receptor; (TKIs): Tyrosine Kinase Inhibitors.

## Competing interests

The authors declare that they have no competing interests.

## Authors' contributions

LC: FISH analysis, conception, and design of the study; MC and PF: technical execution; TV: critical revision of the data and the manuscript; MR: revision of the manuscript.

All authors read and approved the final manuscript.
